# Early Death and Survival of Patients With Acute Promyelocytic Leukemia in ATRA Plus Arsenic Era: A Population-Based Study

**DOI:** 10.3389/fonc.2021.762653

**Published:** 2021-11-16

**Authors:** Hong-Hu Zhu, Ya-Fang Ma, Kang Yu, Gui-Fang Ouyang, Wen-Da Luo, Ren-Zhi Pei, Wei-Qun Xu, Hui-Xian Hu, Shu-Ping Mo, Xiao-Hua Xu, Jian-Ping Lan, Jian-Ping Shen, Li-Hong Shou, Shen-Xian Qian, Wei-Ying Feng, Pu Zhao, Jin-Hong Jiang, Bei-Li Hu, Jin Zhang, Su-Ying Qian, Gong-Qiang Wu, Wen-Ping Wu, Lei Qiu, Lin-Jie Li, Xiang-Hua Lang, Sai Chen, Li-Li Chen, Jun-Bin Guo, Li-Hong Cao, Hui-Fang Jiang, Yong-Ming Xia, Jing Le, Jian-Zhi Zhao, Jian Huang, Yue-Feng Zhang, Ya-Li Lv, Jing-Sheng Hua, Yong-Wei Hong, Cui-Ping Zheng, Ju-Xiang Wang, Bin-Fei Hu, Xiao-Hui Chen, Li-Ming Zhang, Shi Tao, Bing-Shou Xie, Yue-Min Kuang, Wen-Ji Luo, Ping Su, Jun Guo, Xiao Wu, Wei Jiang, Hui-Qi Zhang, Yun Zhang, Chun-Mei Chen, Xiao-Feng Xu, Yan Guo, Jin-Ming Tu, Shao Hu, Xiao-Yan Yan, Chen Yao, Yin-Jun Lou, Jie Jin, the APL Cooperative Group of Zhejiang Province

**Affiliations:** ^1^ Department of Hematology, The First Affiliated Hospital, College of Medicine, Zhejiang University, Hangzhou, China; ^2^ Key Laboratory of Hematologic Malignancies, Diagnosis and Treatment, Hangzhou, China; ^3^ Zhejiang University Cancer Center, Hangzhou, China; ^4^ Zhejiang Laboratory for Systems & Precision Medicine, Zhejiang University Medical Center, Hangzhou, China; ^5^ Department of Hematology, The First Affiliated Hospital of Wenzhou Medical University, Hangzhou, China; ^6^ Department of Hematology, Ningbo First Hospital, Ningbo, China; ^7^ Department of Hematology, Taizhou Hospital of Zhejiang Province affiliated to Wenzhou Medical University, Taizhou, China; ^8^ Department of Hematology, The Affiliated People’s Hospital of Ningbo University, Ningbo, China; ^9^ Department of Hematology, The Children’s Hospital Zhejiang University School of Medicine, Hangzhou, China; ^10^ Department of Hematology, Affiliated Jinhua Hospital, Zhejiang University School of Medicine, Jinhua, China; ^11^ Department of Hematology, The Affiliated Hospital of Jiaxing University, Jiaxing, China; ^12^ Department of Hematology, The Second Affiliated Hospital Zhejiang University School of Medicine, Hangzhou, China; ^13^ Department of Hematology, Zhejiang Provincial People’s Hospital, Hangzhou, China; ^14^ Department of Hematology, Zhejiang Provincial Hospital of Chinese Medicine, Hangzhou, China; ^15^ Department of Hematology, Huzhou Central Hospital, Huzhou, China; ^16^ Department of Hematology, Affiliated Hangzhou First People’s Hospital, Zhejiang University School of Medicine, Hangzhou, China; ^17^ Department of Hematology, Shaoxing People’s Hospital, Wenzhou, China; ^18^ Department of Hematology, Ruian People’s Hospital, Wenzhou, China; ^19^ Department of Hematology, Lishui City People’s Hospital, Lishui, China; ^20^ Department of Hematology, The Second Affiliated Hospital of Jiaxing University, Jiaxing, China; ^21^ Department of Hematology, Sir Run Run Shaw Hospital (SRRSH) Affiliated with the Zhejiang University School of Medicine, Hangzhou, China; ^22^ Department of Hematology, Hwa Mei Hospital, University of Chinese Academy of Sciences, Ningbo, China; ^23^ Department of Hematology, Dongyang Hospital Affiliated to Wenzhou Medical University, Jinhua, China; ^24^ Department of Hematology, People’s Hospital of Quzhou, Quzhou, China; ^25^ Department of Hematology, Zhoushan Hospital, Zhoushan, China; ^26^ Department of Hematology, Lishui Municipal Central Hospital, Jinhua, China; ^27^ Department of Hematology, The First People’s Hospital of Yongkang, Jinhua, China; ^28^ Department of Hematology, Taizhou Central Hospital (Taizhou University Hospital), Taizhou, China; ^29^ Department of Hematology and Oncology, Taizhou First People’s Hospital (Huangyan Hospital of Wenzhou Medical University), Taizhou, China; ^30^ Department of Hematology and Oncology, The First People’s Hospital of Wenling, Taizhou, China; ^31^ Department of Hematology, Shulan Hospital, Hangzhou, China; ^32^ Department of Hematology, Tongde Hospital of Zhejiang Province, Hangzhou, China; ^33^ Department of Hematology, Rheumatology and Nephrology, Yuyao People’s Hospital, Ningbo University Yangming Affiliated Hospital, Ningbo, China; ^34^ Department of Hematology and Oncology, Ningbo Medical Center Lihuili Hospital, Ningbo, China; ^35^ Department of Hematology, Shaoxing Central Hospital, Shaoxing, China; ^36^ Department of Hematology, The Fourth Affiliated Hospital Zhejiang University School of Medicine, Jinhua, China; ^37^ Department of Hematology, The First People’s Hospital of Yuhang District, Hangzhou, China; ^38^ Department of Hematology, Xinchang People’s Hospital, Shaoxing, China; ^39^ Department of Hematology and Oncology, Taizhou Municipal Hospital, Taizhou, China; ^40^ Department of Hematology, Ningbo Yinzhou No. 2 Hospital, Ningbo, China; ^41^ Department of Hematotherapeutic, Wenzhou Central Hospital Medical Group, Wenzhou, China; ^42^ Department of Hematology and Oncology, The Second Affiliated Hospital and Yuying Children’s Hospital of Wenzhou Medical University, Wenzhou, China; ^43^ Department of Pediatric Hematology, Ningbo Women and Children’s Hospital, Ningbo, China; ^44^ Department of Hematology, The Affiliated Hospital of Hangzhou Normal University, Hangzhou, China; ^45^ Department of Hematology, Zhuji People’s Hospital, Shaoxing, China; ^46^ Department of Hematology, Shaoxing Second Hospital, Shaoxing, China; ^47^ Department of Hematology, Wenzhou People’s Hospital, Wenzhou, China; ^48^ Department of Hematology, Jinhua People’s Hospital, Jinhua, China; ^49^ Department of Hematology, The First People’s Hospital of Xiaoshan District, Hangzhou, China; ^50^ Department of Hematology, Zhejiang Xiaoshan Hospital, Hangzhou, China; ^51^ Department of Hematology and Oncology, The Sencond Affiliated Hospital of Zhejiang University, SAHZU Changxing Branch, Huzhou, China; ^52^ Department of Oncology and Hematology, The Affiliated Hospital of Medical School of Ningbo University, Ningbo, China; ^53^ Department of Hematology, Shangyu People’s Hospital, Shaoxing, China; ^54^ Department of Hematology, The First People’s Hospital of Huzhou, Huzhou, China; ^55^ Department of Hematotherapeutic, Yueqing People’s Hospital, Wenzhou, China; ^56^ Department of Hematotherapeutic, The Second Affiliated Hospital of Zhejiang Chinese Medical University, Hangzhou, China; ^57^ Department of Oncology and Hematology, Hangzhou Red Cross Hospital, Hangzhou, China; ^58^ Department of Hematology, The First People’s Hospital of Pinghu, Jiaxing, China; ^59^ Department of Gastroenterology and Hematology, Longyou People’s Hospital, Quzhou, China; ^60^ Department of Hematology and Oncology, The First Hospital of Ninghai County, Ningbo, China; ^61^ Department of Biostatistics, Peking University Clinical Research Institute, Beijing, China

**Keywords:** acute promyelocytic leukemia, ATRA, arsenic, early death, survival

## Abstract

Most randomized trials for acute promyelocytic leukemia (APL) have investigated highly selected patients under idealized conditions, and the findings need to be validated in the real world. We conducted a population-based study of all APL patients in Zhejiang Province, China, with a total population of 82 million people, to assess the generalization of all-trans retinoic acid (ATRA) and arsenic as front-line treatment. The outcomes of APL patients were also analyzed. Between January 2015 and December 2019, 1,233 eligible patients were included in the final analysis. The rate of ATRA and arsenic as front-line treatment increased steadily from 66.2% in 2015 to 83.3% in 2019, with no difference among the size of the center (≥5 or <5 patients per year, *p* = 0.12) or age (≥60 or <60 years, *p* = 0.35). The early death (ED) rate, defined as death within 30 days after diagnosis, was 8.2%, and the 3-year overall survival (OS) was 87.9% in the whole patient population. Age (≥60 years) and white blood cell count (>10 × 10^9^/L) were independent risk factors for ED and OS in the multivariate analysis. This population-based study showed that ATRA and arsenic as front-line treatment are widely used under real-world conditions and yield a low ED rate and a high survival rate, which mimic the results from clinical trials, thereby supporting the wider application of APL guidelines in the future.

## Introduction

Randomized controlled trials (RCTs) remain the gold standard for establishing the efficacy of new cancer therapies under controlled conditions and provide strong evidence that can be used when developing guidelines (1–4). However, the patients enrolled in these trials make up a small and highly selected fraction of the real-world population (5, 6). Therefore, it is very important to know how well and to what extent such guidelines can be generalized to real-world practice.

Acute promyelocytic leukemia (APL) is a unique example of the success of targeted treatment, with a cure rate over 90% in most recent trials (7–17). Based on these series of clinical trials, all-trans retinoic acid (ATRA) plus arsenic trioxide (ATO) as the front-line treatment has been adopted in all contemporary guidelines since 2013 (18–21). A chemotherapy-free treatment using only ATRA and ATO in non-high-risk patients is simple to apply in clinical practice. A completely oral and chemotherapy-free model using oral arsenic and ATRA further simplified the procedures and made home-based treatment a reality for more patients (13, 17, 18, 22). However, until now, there has been no population-based study to test the real-world application of the latest guidelines in APL patients in ATRA plus arsenic era.

This population-based study, which was commissioned and predominantly funded by the APL Cooperative Group of Zhejiang, China, comprised all APL patients during the past 5 years in Zhejiang Province. We aimed to evaluate the extent of the application of the guidelines regarding the use of ATRA and arsenic as the first-line treatment for APL and investigated early death (ED) and survival under real-world conditions stratified by center patient volume, patient age, and calendar year.

## Methods

### Study Design and Oversight

This is a retrospective population-based study including all APL patients who were newly diagnosed from January 2015 to December 2019 from among the entire population of Zhejiang Province, China, accounting for 82 million people. We collected the data from the electronic medical records of all the 57 hematology centers in Zhejiang Province to avoid selection bias (**Supplementary Table S1**).

This study was designed by the APL Cooperation Group of Zhejiang, China. The authors collected and analyzed the data, followed up the cases until April 2020, and wrote the first draft of the manuscript. All the authors reviewed and revised the manuscript, had access to all data, and vouched for the completeness and accuracy of the data. The independent ethics committee at each study center approved the study, and the study was performed in accordance with the principles of the Declaration of Helsinki.

### Patients

Eligible patients newly diagnosed with APL were confirmed by t(15;17) translocation *via* conventional karyotyping or fluorescence *in situ* hybridization or the PML-RARA fusion gene by means of reverse-transcriptase polymerase chain reaction (RT-PCR) assay. Age was not considered as an inclusion criterion. Patients with variants of APL other than t(15;17)/PML-RARA were excluded.

### Treatments

**Figure 1:**
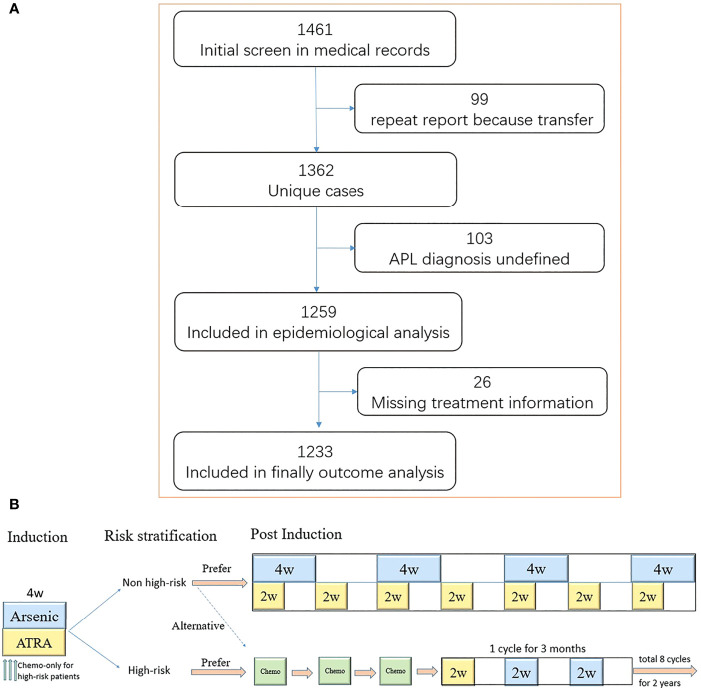
The flow and treatment protocol recommended by guideline 2014. **(A)** Shown is the flow of screening, exclusion, and retention. A total of 99 patients transferred to at least two medical centers. Moreover, 103 patients did not have a definitive diagnosis [11 patients did not perform bone marrow puncture examination, including four early deaths; four patients had a typical acute promyelocytic leukemia (APL) morphology, but the karyotype and molecular biology was negative; 88 patients had a typical APL morphology without karyotype and molecular biology results, including 15 early deaths]. The treatment information of 26 patients was missing. **(B)** Induction therapy lasts for at least 4 weeks until complete remission is achieved. The chemotherapy drugs included anthracycline, cytarabine, and homoharringtonine. One single agent was usually used as induction chemotherapy regimen.

Based on the study on APL0406 published in 2013 that led to changes in clinical practice, ATRA plus ATO as the first-line treatment for APL was first adopted in the National Comprehensive Cancer Network (NCCN) guidelines and China APL guidelines (ATRA plus ATO or oral arsenic) in 2014 and has continued to be included in subsequent versions (**Figure 1**) (19, 20).

For low- and intermediate-risk patients, ATRA (25 mg/m² daily) plus ATO (0.15 mg/kg daily) or Realgar-Indigo naturalis formula (RIF) (60 mg/kg daily) is recommended for at least 4 weeks as induction therapy. The consolidation therapy included ATO (0.15 mg/kg daily) or RIF (60 mg/kg daily) in a 4-week-on/4-week-off regimen for four cycles and ATRA (25 mg/m² daily) in a 2-week-on/2-week-off regimen for seven cycles for approximately 7 months (13, 17, 19, 20). High-risk patients are recommended to receive ATRA (25 mg/m² daily) plus ATO (0.15 mg/kg daily) or RIF (60 mg/kg daily) for at least 4 weeks in addition to chemotherapy as induction therapy. The consolidation therapy consisted of three cycles of chemotherapy. The maintenance treatment included sequential ATRA, followed by either RIF or ATO for 2 years (12).

ATRA plus ATO or oral arsenic was shortened to ATRA/arsenic, and ATRA plus anthracycline-based chemotherapy was shortened to ATRA/anthracycline in the following.

### Definition and Endpoints

The risk category was based on the Sanz criteria (10). ED was defined as death within 30 days after diagnosis of APL. Complete remission (CR), overall survival (OS), and cumulative incidence of relapse (CIR) were defined according to the National Cancer Institute workshop definitions (23). Event-free survival (EFS) was defined as the time from diagnosis to failure to achieve CR, relapse, or death, whichever occurred first. The centers were divided into large (≥5 APL patients per year) and small (<5 APL patients per year) groups.

The primary endpoint of this study was the rate of ATRA/arsenic as the first-line treatment in the real world. The secondary endpoints included ED, CR, CIR, EFS, and OS.

### Statistical Analysis

The survival functions were estimated using the Kaplan–Meier method and were compared using the log-rank test. Dichotomous variables were compared with Fisher’s exact test or the chi-square test, and continuous variables were compared with the Wilcoxon rank sum test. Multivariate analysis was performed using binary logistic regression model or Cox proportional hazards regression model, for which variables with prognostic potential from the univariate analysis were involved. All statistical tests were two-tailed, with a significance level of 0.05. Our analysis used all participants for whom the variables of interest were available. The data were analyzed using SAS software version 9.4 (SAS Institute, Cary, NC).

We calculated the ED rate, the CR rate, and the estimated survival rate of all patients. As regards the therapeutic regimens, we compared ATRA/arsenic with ATRA/anthracycline as first-line treatment. When we compared these two mainly therapeutic regimens, patients who died before anti-leukemia treatment or with monotherapy were ruled out. To reduce the confounding bias between the two groups, propensity score was estimated based on three clinical variables, including gender, age, and risk category. Logistic regression was applied to create a continuous propensity score ranging from 0 to 1. The matched ATRA/arsenic and ATRA/anthracycline groups were generated by one-to-four data matching with a caliper of 0.1 standard deviation.

## Results

### Patient Characteristics and APL Incidence

We initially identified 1,461 patients from the medical database and finally included 1,233 patients with newly diagnosed APL (652 male and 581 female patients) with a median age of 44 years (range, 1–91 years) (**Figure 1** and **Table 1**). The distribution and number of patients in each center are shown in **Supplementary Table S1**. Age followed a normal distribution, as 117 (9.5%) patients were <20 years old and 234 (19.0%) patients were ≥60 years old (**Supplementary Figure S1**). The annual incidences of APL during the years from 2015 to 2019 were 0.28, 0.28, 0.29, 0.32, and 0.32 per 100,000, respectively (**Supplementary Table S2**), and the average annual incidence of APL was 0.30 per 100,000.

**Table 1:** Characteristics of the patients at diagnosis.

Variable	
Age	
Median (range), year	44 (1–91)
Distribution, number (%)	
0 to 19 years	117 (9.5)
20 to 39 years	373 (30.3)
40 to 59 years	509 (41.3)
≥60 years	234 (19.0)
Sex, number (%)	
Male	652 (52.9)
Female	581 (47.1)
Risk, number (%)	
Low	364 (29.6)
Intermediate	578 (47.0)
High	289 (23.4)
10 × 10^9^/L<WBC ≤50 × 10^9^/L	210 (17.0)
50 × 10^9^/L<WBC ≤100 × 10^9^/L	57 (4.6)
WBC>100 × 10^9^/L	22 (1.8)
PML-RARA isoform, number (%)	
Long	710 (57.6)
Short	375 (30.4)
Variant	39 (3.2)
Unknown	109 (8.8)
Size of medical center, number of centers (%), number of patients (%)	
Small	45 (78.9), 467 (37.9)
Large	12 (21.1), 766 (62.1)

A total of 957 patients received ATRA/arsenic as the first-line treatment, and 524 of them were matched with 131 patients in the ATRA/anthracycline group by propensity score match. Of the remaining 145 patients, 37 died early before receiving ATRA/arsenic or ATRA/anthracycline regimen, and the other 108 received regimens such as ATRA plus homoharringtonine, anthracycline plus cytarabine, and so on.

### ATRA/Arsenic as First-Line Treatment

**Figure 2:**
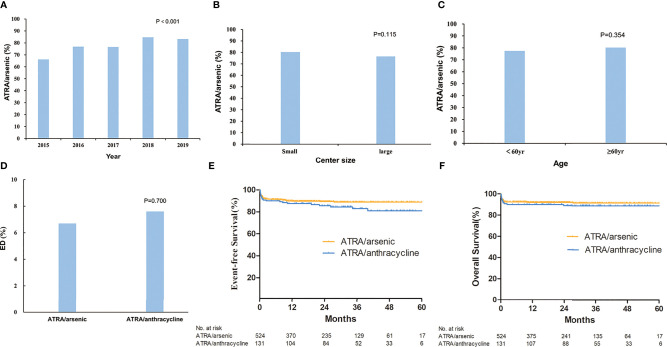
The rate of all-trans retinoic acid (ATRA)/arsenic as first-line treatment for acute promyelocytic leukemia and outcomes. Shown is the rate of ATRA/arsenic as first-line treatment which changed with the alteration of year **(A)**, center size **(B)**, and age **(C)**. The early death rate **(D)** and estimated survival **(E, F)** were compared between ATRA/arsenic group and ATRA/anthracycline group.

The total rate of ATRA/arsenic as the first-line treatment during the 5 years was 78.0%. We further assessed the changes in the rate of ATRA/arsenic as the first-line treatment among calendar years, centers with different patient volumes, and patient ages (**Figure 2**). The rate of ATRA/arsenic as the first-line treatment increased steadily from 66.2% in 2015 to 83.3% in 2019 (*p* < 0.001), and patients receiving postremission treatment with chemotherapy decreased from 78.4 to 36.3%. The proportion of patients receiving home-based postremission treatment with oral arsenic and ATRA increased from 16.8% in 2015 to 55.9% in 2019.

Moreover, we found no difference in the rate of ATRA/arsenic as the first-line treatment among centers of different sizes (≥5 or <5 patients per year, *p* = 0.12) and patients of different ages (≥60 or <60 years, *p* = 0.35).

### ED

**Figure 3:**
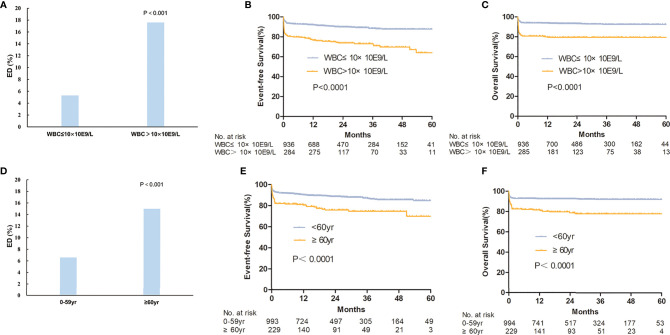
The survival between different risk groups and ages. The early death rate **(A)** and estimated survival **(B, C)** were compared between high-risk group (WBC>10×10E9/L) and non-high-risk group (WBC≤10×10E9/L). The early death rate **(D)** and estimated survival **(E, F)** were also compared between patients ≥60 years and <60 years.

The overall ED rate was 8.2% and did not decline significantly from 2015 to 2019 (7.60, 7.00%, 8.40, 5.60, and 12.00%, *p* = 0.085) (**Supplementary Figure S2 **and** Supplementary Table S3**). Furthermore, 1.5% of patients died between 30 to 60 days. The cause of ED included the following: intracranial hemorrhage (*n* = 61, 59.4%), pulmonary hemorrhage (*n* = 6, 5.9%), serious infection (*n* = 14, 13.9%), organ failure or cardiac arrest (*n* = 14, 13.9%), cerebral infarction (*n* = 3, 3.0%), and others (*n* = 3, 3.0%). In the early dead, most patients (*n* = 54, 53.5%) received ATRA/arsenic as the first-line treatment, 36 patients (35.6%) received ATRA or arsenic ± chemotherapy, nine patients (8.9%) died before any treatment, and the last two patients (2.0%) underwent chemotherapy without ATRA or arsenic. The ED rate was not different between small and large centers (9.2 and 7.6%, *p* = 0.310). The ED rate was higher in male patients (9.7 *vs*. 6.5%, *p* = 0.046), older patients (4.3% for <20 years, 5.6% for 20–39 years, 7.9% for 40–59 years, and 15.0% for ≥60 years; *p* < 0.001), high-risk patients (17.6% in high-risk patients, 6.7% in intermediate-risk patients, and 3.0% in low-risk patients, *p* < 0.001) (**Figure 3** and **Supplementary Figure S2**). For high-risk patients, the ED rate increased with increasing WBC count (12.9% for 10–50 × 10^⁹^/L, 31.6% for 50–100 × 10^⁹^/L, 27.3% for >100 × 10^⁹^/L; *p* < 0.001, **Supplementary Figure S2**). **Supplementary Table S4** shows all the population-based results regarding ED in APL patients in the literature and the present study. Age (≥60 years) and WBC count (>10 × 10^9^/L) were independent risk factors for ED in the multivariate analysis (**Table 2**). The ED rate of ATRA/arsenic group and ATRA/anthracycline group had no significant difference (6.7 *vs*. 7.6%; *p* = 0.700, **Figure 2**).

**Table 2:** Univariate and multivariate analyses of early death, event-free survival, and overall survival.

Variable	Early death				Event-free survival				Overall survival			
	Univariate		Multivariate		Univariate		Multivariate		Univariate		Multivariate	
	OR (95% CI)	*P*-value	OR (95% CI)	4.1.1 *P*-value	HR (95% CI)	4.1.2 *P*-value	HR (95% CI)	4.1.3 *P*-value	HR (95% CI)	4.1.4 *P*-value	HR (95% CI)	4.1.5 *P*-value
Gender, male *vs*. female	1.528 (1.005–2.324)	0.047	1.479 (0.958–2.283)	0.077	1.512 (1.119–2.044)	0.007	1.433 (1.058–1.941)	0.020	1.415 (0.999–2.005)	0.051	1.396 (0.984–1.982)	0.062
Age (≥60 *vs*. <60 years)	2.486 (1.605–3.851)	<0.001	3.374 (2.114–5.386)	<0.001	2.221 (1.622–3.042)	<0.001	2.834 (2.055–3.909)	<0.001	2.867 (2.021–4.068)	<0.001	3.730 (2.602–5.345)	<0.001
WBC (>10 × 10^9^/L *vs*. ≤10×10^9^/L)	3.823 (2.523–5.792)	<0.001	4.504 (2.910–6.970)	<0.001	2.940 (2.191–3.946)	<0.001	3.078 (2.265–4.181)	<0.001	3.117 (2.217–4.383)	<0.001	3.757 (2.648–5.331)	<0.001
PLT (>40 × 10^9^/L *vs*. ≤40 × 10^9^/L)	0.621 (0.389–0.992)	0.046	0.769 (0.471–1.255)	0.293	0.575 (0.407–0.813)	0.002	0.691 (0.485–0.985)	0.041	0.628 (0.424–0.931)	0.021	0.759 (0.506–1.137)	0.181
Arsenic in introduction (iv *vs*. oral)	3.991 (0.544–29.305)	0.174			2.264 (0.719–7.127)	0.163			2.622 (0.645–10.661)	0.178		
Treatment regimen, ATRA/arsenic *vs*. ATRA/anthracycline	0.866 (0.417–1.798)	0.700			0.723 (0.439–1.192)	0.204			0.838 (0.461–1.525)	0.563		
Size of centers (large *vs*. small)	0.808 (0.535–1.220)	0.310			0.829 (0.616–1.117)	0.219			0.763 (0.542–1.075)	0.122		
Calendar												
**4.1.6 ** 2016 *vs*. 2015	0.906 (0.446–1.841)	0.785			0.827 (0.512–1.334)	0.436			0.958 (0.537–1.707)	0.883		
**4.1.7 ** 2017 *vs*. 2015	1.107 (0.564–2.171)	0.768			1.043 (0.658–1.653)	0.859			1.037 (0.589–1.828)	0.899		
**4.1.8 ** 2018 *vs*. 2015	0.724 (0.353–1.485)	0.379			0.839 (0.515–1.366)	0.480			0.830 (0.462–1.492)	0.533		
**4.1.9 ** 2019 *vs*. 2015	1.652 (0.894–3.053)	0.109			1.349 (0.853–2.133)	0.200			1.549 (0.921–2.605)	0.099		

It is worth noting that a definitive diagnosis of APL was not made in 103 patients, and they were excluded from the analysis. Thirty of these patients died early, and 16 of them were lost to follow-up due to transfer or going home.

### CR

The overall CR rate was 90.3% (95% CI, 88.6–91.9%, **Supplementary Table S3**). The CR rate was higher in females (92.2 *vs*. 88.5%, *p* = 0.034), young patients (94.0% for <20 years, 93.8% for 20–39 years, 91.1% for 40–59 years, and 81.0% for ≥60 years; *p* < 0.001), and low-risk patients (80.8% in high-risk patients, 91.8% in intermediate-risk patients, and 95.3% in low-risk patients; *p* < 0.001) than in their counterparts. For high-risk patients, the CR rate decreased with increasing WBC count (85.6% for 10–50 × 10^⁹^/L, 66.7% for 50–100 × 10^⁹^/L, 71.4% for >100 × 10^⁹^/L; *p* = 0.003). The CR rate of the ATRA/arsenic group and the ATRA/anthracycline group had no significant difference (91.5 *vs*. 91.5%; *p* = 1.000).

### Survival and Relapse

The 3-year OS, EFS, and CIR probabilities were 87.9% (95% CI, 85.2–90.1%), 84.8% (95% CI, 82.4–86.9%), and 5.6% (95% CI, 1.9–12.3%) (**Supplementary Table S3**), respectively. The rate of relapse in central nervous system leukemia was 2.1%, and there was no association with risk level, age, or treatment regimen. The CNS relapse rate had no statistical difference between three risk groups (1.2% for low-risk patients, 1.8% for intermediate-risk patients, and 4.0% for high-risk patients; *p* = 0.053). The CNS relapse rate of the ATRA/arsenic group and the ATRA/anthracycline group also had no significant difference (1.5 *vs*. 2.7%; *p* = 0.421). Age (≥60 years) and WBC count (>10 × 10^9^/L) were independent risk factors for EFS and OS in the multivariate analysis (**Table 2** and **Figure 3**).

No differences in EFS and OS existed between large and small centers or between ATO and oral arsenic. The EFS and OS of the ATRA/arsenic group and the ATRA/anthracycline group also had no statistical difference (3-year EFS: 88.9 *vs*. 82.9%, *p* = 0.124; 3-year OS: 91.5 *vs*. 88.9%, *p* = 0.387) (**Figure 2**). **Supplementary Table S4** shows all the population-based results regarding the long-term OS of APL patients in the literature and the present study.

Age stratification had no statistical effect on the cumulative incidence of relapse. Under different risk categories, the CIR had significant statistical difference (**Supplementary Figure S3**). The CIR was higher in high-risk patients than that in intermediate-risk patients (3-year CIR: 9.7 *vs*. 6.1%, *p* = 0.028), while it had no significant statistical difference between intermediate- and low-risk categories (3-year CIR: 6.1 *vs*. 3.2%, *p* = 0.101). In the ATRA/arsenic group, the 3-year CIR of high-risk categories and intermediate-risk categories was 7.0 and 4.4% (*p* = 0.029), while the 3-year CIR of intermediate-risk categories and low-risk categories was 4.4 and 3.0% (*p* = 0.237). Lower CIR was observed in the ATRA/arsenic group than in ATRA/anthracycline group (3-year CIR: 2.5 *vs*. 7.9%, *p* = 0.027).

## Discussion

In this large population-based study, we obtained direct evidence of how well and to what extent the latest guidelines for APL, which were based on the results of clinical trials, are applied under real-world conditions. More than 80% of patients in not only large centers but also small centers received the treatment recommended by the guidelines. Moreover, current treatments mainly with ATRA and arsenic as the first-line treatment resulted in a low ED rate of 8.2% and an improved long-term survival rate of 88%, which mimic the results of clinical trials, suggesting the generalizability and wide applicability of the APL guidelines. More than half of the patients received chemotherapy-free, home-based postremission treatment, which meets the medical needs of the patients, especially during emergency situations such as the COVID-19 pandemic.

Although many clinical trials have provided convincing evidence and their findings have been incorporated into the latest guidelines, few studies have assessed the outcome of the application of those guidelines in the real world. For tumors, theoretically, a chemotherapy-free and home-based treatment is most likely to result in excellent adherence. The latest guidelines for APL in China recommend the use of ATRA plus oral arsenic without chemotherapy, and home-based postremission therapy is recommended for first-line treatment, leading to excellent adherence to the guidelines in all hospitals. The rate of ATRA/arsenic as the first-line treatment increased steadily from 66.2% in 2015 to 83.3% in 2019, and the proportion of patients receiving chemotherapy-containing postremission treatment decreased from 78.4 to 36.3%.

The greatest obstacle to curing all APL patients is the high ED rate, according to most population-based studies. The ED rate was approximately 17.2–29% and did not significantly decrease in the ATRA plus chemotherapy era (24–29). A population-based study of whether the ED rate can be reduced in the ATRA plus arsenic era has been needed (30), especially since the NCCN guidelines for APL were published in 2013. Our study, for the first time, provides strong evidence that the ED rate can be significantly reduced to 8.2% or lower in ATRA plus arsenic era in a population-based study, which is also supported by recent results with ED rates of 4.8–6.8% in some single-center studies from China (31, 32). Moreover, the lower ED rate translated into a higher 3-year OS rate of 87.9% in our study. However, the ED rate may be underestimated. A total of 30 patients died within a short time before confirming the diagnosis, and some of them may be diagnosed with APL. The use of low-dose or no chemotherapy during induction therapy reduced the related mortality, and physicians had adequate experience with APL treatment (29). In China, hematologists must receive continued medical education in large centers, and information on APL has been compulsory content in recent years, promoting the recognition and treatment of APL as early as possible. The well-informed focus on APL has resulted in a relatively small difference in the ED rate between large and small centers. The relatively high ED rate among patients with high WBC counts above 10 × 10^⁹^/L or old age over 60 years has become the last obstacle on the road to achieving a cure for all patients, which was also demonstrated in previous population-based studies pre-ATRA plus ATO era or a single-center study in ATRA plus ATO era (24, 25, 30–32).

The ATRA dose was 25 mg/m^2^ in divided doses daily in our study, smaller than the recommended 45 mg/m^2^ in the NCCN guideline and ELN guideline. This ATRA dose is recommended in China APL guidelines (19, 20). A study first demonstrated that there was no difference in terms of therapeutic efficacy, triggering of hyperleukocytosis, or retinoic acid syndrome and pharmacokinetic results with ATRA at 25 or 45 mg/m^2^/day (33). Another clinical trial showed that low-dose ATRA (15–20 mg/m^2^) is as effective as the high-dose one (45 mg/m^2^/day) in treating APL patients and may provide advantages through decreased hyperleukocytosis and other side effects (34). Moreover, most recent clinical trials in China, including ATRA dose of 25 mg/m^2^ daily as front-line treatment of APL, showed good molecular remission and survival (12, 13, 17, 35).

Under real-world conditions, the OS improved, reaching 91.7% when ATRA plus arsenic was used as the front-line treatment, which is another key finding in our study. Previous population-based studies showed that the OS ranged from 54.6 to 68% (24–28). The higher OS in our study may be partially explained by the low ED rate and decreased relapse rate. Arsenic plus ATRA was shown to significantly reduce the relapse rate compared to ATRA plus chemotherapy in recent clinical trials (11, 16, 36). This was consistent with our study. The 3-year CIR was as low as 2.3% of the ATRA/arsenic group compared with 7.9% of the ATRA/anthracycline group. In addition, the CIR was higher in high-risk categories than that in intermediate-risk categories, and it had no statistical difference between intermediate- and low-risk categories in our study. Similar results were obtained in the APL2012 trial (35).

There are some limitations of our study. First, some patients who died within a short time at home or at the hospital without providing marrow samples were excluded from the analysis, which may have led to the underestimation of the ED rate. Second, frequent migration within China could have caused some local residents to be diagnosed and treated outside of Zhejiang Province or new patients from other provinces to be diagnosed and treated in Zhejiang Province, potentially biasing the exact number of new cases of APL. Third, the retrospective nature of the study may have resulted in missing information, potentially preventing further analysis. We are currently conducting a prospective real-world study of APL (ChiCTR1900023308).

In summary, we herein provide evidence that the APL guidelines have been extensively applied in real-world clinical practice, resulting in low ED rate, low relapse rate, and improved survival rate. The goal of achieving a cure for all APL patients is attainable, and the best summary of the successful treatment strategy is “the simpler, the better”.

## Data Availability Statement

The original contributions presented in the study are included in the article/**Supplementary Material**. Further inquiries can be directed to the corresponding author.

## Ethics Statement

The studies involving human participants were reviewed and approved by the Clinical Research Ethics Committee—the IIT Ethics Review Group, the First Affiliated Hospital of Zhejiang University Medical College. Written informed consent to participate in this study was provided by the legal guardian/next-of-kin of the participants.

## Author Contributions

H-HZ, Y-FM, and JJ contributed to the study design and were responsible for the analysis and interpretation of the data as well as drafting and critical reviewing the manuscript. KY, G-FO, W-DL, R-ZP, W-QX, H-XH, S-PM, X-HX, J-PL, J-PS, L-HS, S-XQ, W-YF, PZ, J-HJ, B-LH, JZ, S-YQ, G-QW, W-PW, LQ, L-JL, X-HL, SC, L-LC, J-BG, L-HC, H-FJ, Y-MX, JL, J-ZZ, JH, Y-FZ, Y-LL, J-SH, Y-WH, C-PZ, J-XW, B-FH, X-HC, L-MZ, ST, B-SX, Y-MK, W-JL, PS, JG, XW, WJ, H-QZ, YZ, C-MC, X-FX, YG, J-MT, SH, and Y-JL contributed to data collection. X-YY and CY analyzed and interpreted the data. All authors contributed to the article and approved the submitted version.

## Funding

This work was supported by the National Natural Science Foundation of China (81970133 to H-HZ and 81820108004 to JJ) and the Zhejiang Province Leading Innovation Team Project (2020R01006 to H-HZ).

## Conflict of Interest

The authors declare that the research was conducted in the absence of any commercial or financial relationships that could be construed as a potential conflict of interest.

## Publisher’s Note

All claims expressed in this article are solely those of the authors and do not necessarily represent those of their affiliated organizations, or those of the publisher, the editors and the reviewers. Any product that may be evaluated in this article, or claim that may be made by its manufacturer, is not guaranteed or endorsed by the publisher.
