# Supplementary material for: Early Death and Survival of Patients With Acute Promyelocytic Leukemia in ATRA Plus Arsenic Era: A Population-Based Study
Source: Front Oncol. 2021 Nov 16;11:762653. doi: 10.3389/fonc.2021.762653 (PMC8637823; doi:10.3389/fonc.2021.762653)
Supplement: Supplementary file 1 [file DataSheet_1.docx]

**Supplementary Appendix**

**Table of Contents**

**SUPPLEMENTARY FIGURES**

Figure S1. The age distribution among all the APL patients2

Figure S2. The early death 3

Figure S3. The cumulative incidence of relapse 4

Figure S4. Survival of all the APL atients 5

**SUPPLEMENTARY TABLES**

Table S1. The centers and number of APL patients included in the study 6

Table S2. The population-based data in Zhejiang Province 8

Table S3. Outcomes 8

Table S4. The population-based data of APL from literature and the present study 9

Reference 9


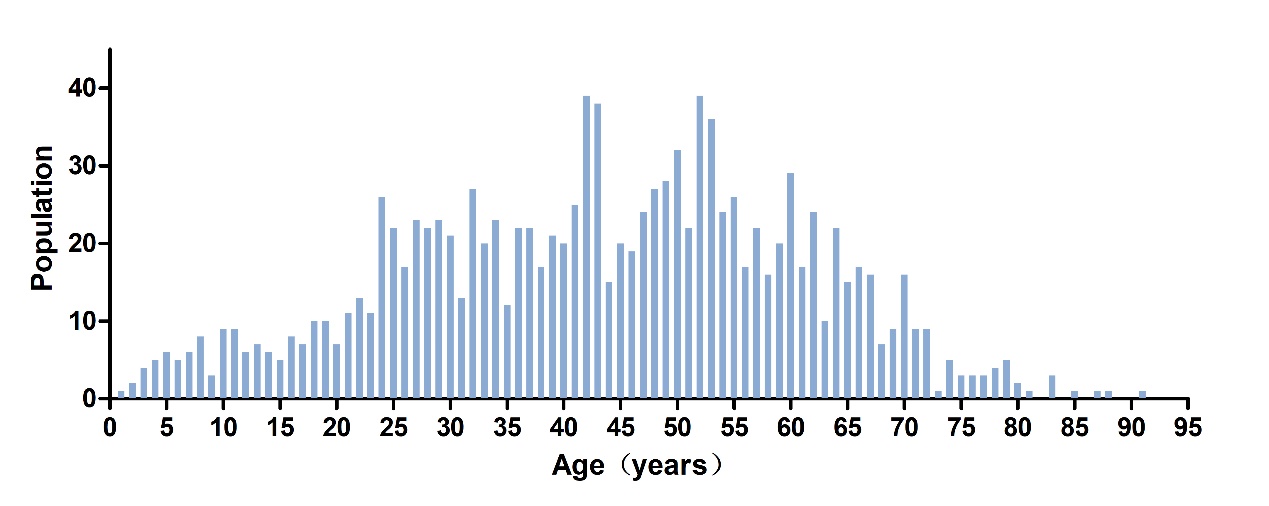


**Figure S1. The age distribution among all the APL patients**


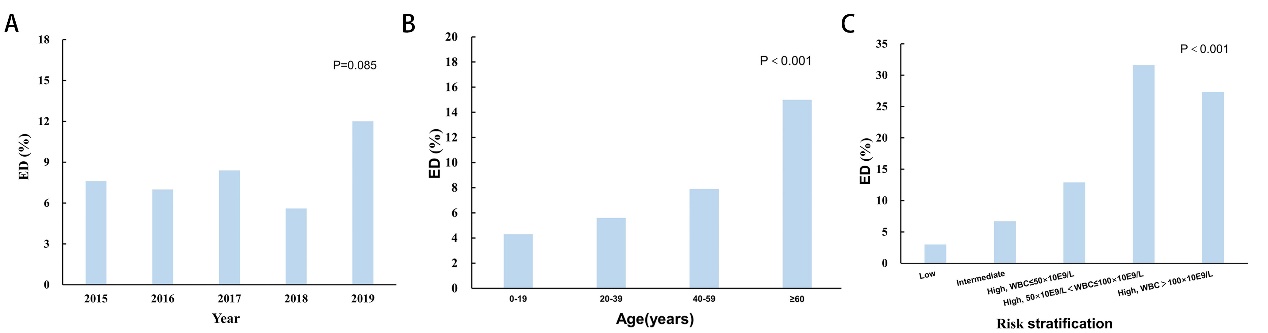


**Figure S2. The early death**


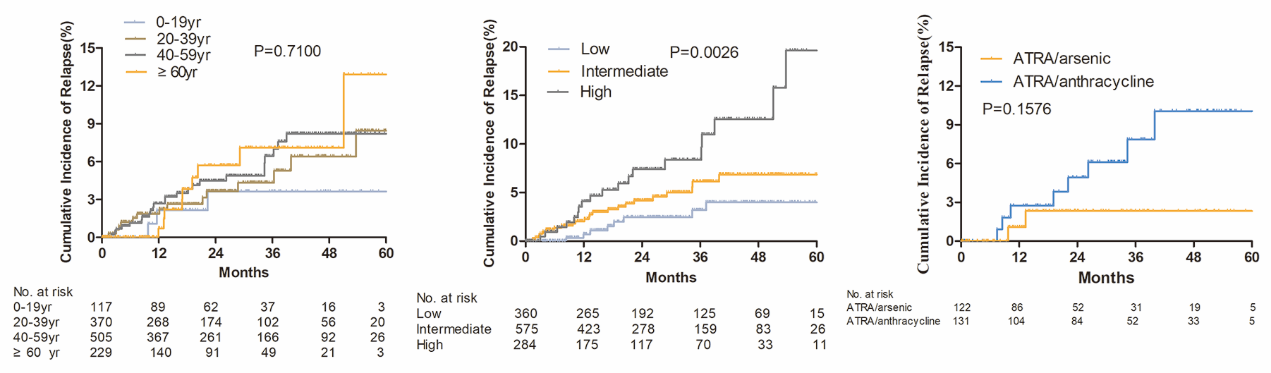


**Figure S3. The cumulative incidence of relapse**


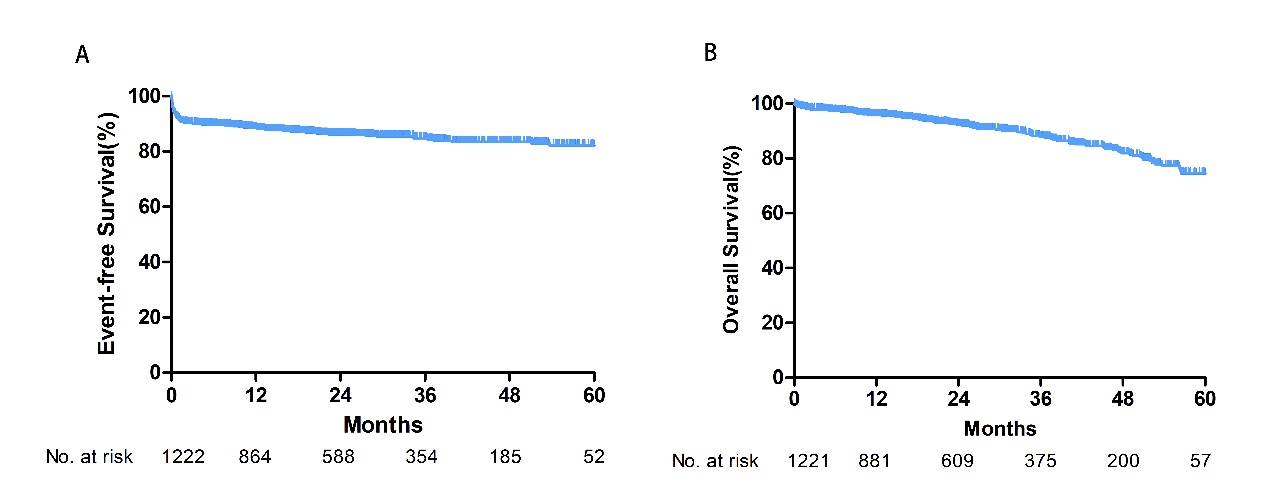


**Figure S4. Survival of all the APL patients.**

| Table S1. The centers and number of APL patients included in the study | |
| --- | --- |
| Medical center | **Number of cases^ψ^** |
| The First Affiliated Hospital, College of Medicine, Zhejiang University | 282 |
| The First Affiliated Hospital of Wenzhou Medical University | 89 |
| Ningbo First Hospital | 62 |
| Taizhou Enze Medical Center Taizhou Hospital | 55 |
| The Affiliated People's Hospital of Ningbo University | 50 |
| The Children's Hospital Zhejiang University School of Medicine | 50 |
| The Second Affiliated Hospital Zhejiang University School of Medicine | 38 |
| The Affiliated Hospital of Jiaxing University | 35 |
| Affiliated Jinhua Hospital, Zhejiang University School of Medicine | 31 |
| Affiliated Hangzhou First People's Hospital, Zhejiang University School of Medicine | 25 |
| Ruian People's Hospital | 25 |
| Lishui City People's Hospital | 25 |
| Shaoxing People's Hospital | 23 |
| Zhejiang Provincial People's Hospital | 21 |
| Hwa Mei Hospital, University of Chinese Academy of Sciences | 21 |
| Zhejiang Provincial Hospital of Chinese Medicine | 20 |
| The Second Affiliated Hospital of Jiaxing University | 20 |
| People's Hospital of Quzhou | 20 |
| Huzhou Central Hospital | 19 |
| Dongyang Hospital Affiliated to Wenzhou Medical University | 19 |
| The First People's Hospital of Yongkang | 17 |
| Lishui Municipal Central Hospital | 17 |
| Taizhou Central Hospital (Taizhou University Hospital) | 17 |
| Sir Run Run Shaw Hospital (SRRSH), Affiliated with the Zhejiang University School of Medicine | 16 |
| Taizhou First People's Hospital (Huangyan Hospital of Wenzhou Medical University) | 16 |
| Zhoushan Hospital | 15 |
| The First People's Hospital of Wenling | 15 |
| Yuyao People's Hospital, Ningbo University Yangming Affiliated Hospital | 13 |
| Tongde Hospital of Zhejiang Province | 11 |
| Xinchang People's Hospital | 11 |
| Shaoxing Central Hospital | 10 |
| The Fourth Affiliated Hospital Zhejiang University School of Medicine | 10 |
| The First People's Hospital of Yuhang District, Hangzhou | 10 |
| Taizhou Municipal Hospital | 9 |
| The Second Affiliated Hospital and Yuying Children's Hospital of Wenzhou Medical University | 9 |
| The Affiliated Hospital of Hangzhou Normal University | 8 |
| Zhuji People's Hospital | 8 |
| Ningbo Medical Center Lihuili Hospital | 7 |
| Wenzhou Central Hospital Medical Group | 7 |
| Shulan (Hangzhou) Hospital | 6 |
| Ningbo Yinzhou No.2 Hospital | 6 |
| Ningbo Women and Children's Hospital | 6 |
| Shaoxing Second Hospital | 6 |
| Shangyu People's Hospital | 6 |
| Wenzhou People's Hospital | 5 |
| Jinhua People's Hospital | 5 |
| Zhejiang Xiaoshan Hospital | 5 |
| SAHZU.Changxing Campus | 5 |
| The First People's Hospital of Xiaoshan District, Hangzhou | 4 |
| The Second Affiliated Hospital Of Zhejiang Chinese Medical University | 4 |
| Hangzhou Red Cross Hospital | 4 |
| The First People's Hospital of Pinghu | 4 |
| The Affiliated Hospital of Medical School of Ningbo University | 3 |
| The First People's Hospital of Huzhou | 2 |
| Yueqing People's Hospital | 2 |
| Longyou People's Hospital | 2 |
| The First Hospital of Ninghai County | 2 |

**ψ Repeated cases were counted only in medical centers where they primary diagnosed.**

| Table S2. The population-based data in Zhejiang Province^δ^ | | | | | |
| --- | --- | --- | --- | --- | --- |
| Year | **No. of new cases** | **Resident population (million)** | **Flowing population (million)** | **Total population (million)** | **Annual incidences**  **(per 100,000)** |
| 2015 | 223 | 55.39 | 22.86 | 78.26 | 0.28 |
| 2016 | 230 | 55.90 | 25.15 | 81.05 | 0.28 |
| 2017 | 239 | 56.57 | 26.10 | 82.67 | 0.29 |
| 2018 | 266 | 57.37 | 26.73 | 84.10 | 0.32 |
| 2019 | 275 | 58.50 | 27.73 | 86.23 | 0.32 |

δ Patients diagnosed and treated in medical centers in Zhejiang Province were included in the study, no matter where their native place were.

| Table S3. Outcomes | | |
| --- | --- | --- |
| Outcomes | **(%)** | **95% CI (%)** |
| 30-day mortality | 8.2 | 6.7-9.7 |
| Complete remission rate | 90.3 | 88.6-91.9 |
| 3-year cumulative incidence of relapse | 5.6 | 1.9-12.3 |
| 3-year event-free survival | 84.8 | 82.4-86.9 |
| 3-year overall survival | 87.9 | 85.2-90.1 |

| Table S4. The population-based data of APL from literature and the present study | | | | | | | | | | |
| --- | --- | --- | --- | --- | --- | --- | --- | --- | --- | --- |
| Authors | **published time** | **Country** | **Resource of data** | **Calendar period** | **Total No.** | **Age**  **(median,range)** | **Incidence**  **(per100000)** | **Front-line Treatment** | **ED (%)** | **OS(%)** |
| Lehmann S, et al.^1^ | 2011 | Swedish | population-based | 1997-2006 | 105 | 52(18-86) | 0.145 | ATRA+Chemotherapy | 29.0 | 62(6y) |
| Park JH, et al.^2^ | 2011 | USA | population-based | 1992-2007 | 1400 | 44(all age) | 0.23 | ATRA+Chemotherapy | 17.3 | 65.7(3y) |
| Paulson K,et al.^3^ | 2014 | Canadian | population-based | 1993-2007 | 399 | 47.9(all age) | 0.083 | ATRA+Chemotherapy | 21.8 | 54.6(5y) |
| Abrahão R, et al.^4^ | 2015 | USA | population-based | 1988–2011 | 772 | 27(0-39) | NA | ATRA+Chemotherapy | 17.2 | 68.1(5y) |
| Dinmohamed AG, et al.^5^ | 2016 | Netherlands | population-based | 1989–2012 | 617 | 52(all age) | 0.15 | ATRA+Chemotherapy | 20.0 | 66(5y) |
| Thuler LCS, et al.^6^ | 2017 | Brazil | population-based | 2001-2012 | 614 | 30(all age) | NA | ATRA+Chemotherapy | 19.4 | NA |
| Zhu HH, et al. | present | China | population-based | 2015-2019 | 1233 | 44(all age) | 0.3 | ATRA+ATO/RIF | 8.2 | 87.8(3y) |

**Reference**

1. Lehmann S, Ravn A, Carlsson L, Antunovic P, Deneberg S, Mollgard L, et al. Continuing high early death rate in acute promyelocytic leukemia: a population-based report from the Swedish Adult Acute Leukemia Registry. Leukemia 2011; 25(7): 1128-1134.

2. Park JH, Qiao B, Panageas KS, Schymura MJ, Jurcic JG, Rosenblat TL, et al. Early death rate in acute promyelocytic leukemia remains high despite all-trans retinoic acid. Blood 2011, 2011; 118(5): 1248-1254.

3. Paulson K, Serebrin A, Lambert P, Bergeron J, Seftel M, et al. Acute promyelocytic leukaemia is characterized by stable incidence and improved survival that is restricted to patients managed in leukaemia referral centres: a pan-Canadian epidemiological study. Br J Haematol. 2014;166(5):660-6.

4. Abrahão R, Ribeiro RC, Medeiros BC, et al. Disparities in early death and survival in children, adolescents, and young adults with acute promyelocytic leukemia in California. Cancer 2015;121(22):3990-7.

5. Dinmohamed AG, Visser O, van Norden Y, Blijlevens NM, Cornelissen JJ, Huls GA,et al.Treatment, trial participation and survival in adult acute myeloid leukemia: a population-based study in the Netherlands, 1989-2012. Leukemia. 2016;30(1):24-31.

6. Thuler LCS, Pombo-de-Oliveira MS.Acute promyelocytic leukaemia is highly frequent among acute myeloid leukaemias in Brazil: a hospital-based cancer registry study from 2001 to 2012.Ann Hematol. 2017;96(3):355-362.
